# Conjunctival inclusion cyst following repair of tube erosion in a child with aphakic glaucoma, leading to endophthalmitis

**DOI:** 10.3205/oc000025

**Published:** 2015-05-22

**Authors:** Avik Kumar Roy, Sirisha Senthil

**Affiliations:** 1 L V Prasad Eye Institute, Kallam Anji Reddy campus, Hyderabad, India

**Keywords:** Ahmed Glaucoma Valve, tube erosion, conjunctival inclusion cyst

## Abstract

**Introduction:** Glaucoma in aphakia is a major long term complication following congenital cataract surgery. Implantation of glaucoma drainage device provides an effective approach to manage refractory paediatric glaucoma. However implant surgery in young individuals is not free of complications. The prompt detection and management of tube erosion is of utmost importance to prevent devastating sequel of endophthalmitis. Implantation cyst following repair of tube erosion has not been reported so far. This case illustrates the rare occurrence of inclusion cyst following repair of tube erosion, the possible causes and its consequences.

**Case description:** A 2-year-old child with aphakia developed intractable glaucoma. Following a failed glaucoma filtering surgery he underwent sequential Ahmed Glaucoma Valve implantation in both the eyes. Six weeks following right eye surgery, the child presented with conjunctival erosion overlying the tube, which was treated with scleral patch graft and conjunctival advancement. One month after the repair of tube erosion, the child presented with implantation cyst under the scleral patch graft, which was treated by drainage with a 29G needle. The child presented with endophthalmitis of his right eye following an episode of bilateral conjunctivitis. This was managed by an emergency pars plana vitrectomy, intraocular antibiotics and tube excision. At the last follow up visit, the IOP was 20 mmHg with 2 topical antiglaucoma medications in the right eye following a trans scleral photocoagulation.

**Discussion:** Lifelong careful follow-up of paediatric eyes with implant surgery is mandatory to look for complication such as tube erosion. It is important to place additional sutures to secure the patch graft during implantation of glaucoma drainage devices in children to prevent graft displacement and consequent tube erosion. During repair of tube erosion, it is crucial to remove all the conjunctival epithelium around the tube, thus not to incorporate epithelial tissue within the surgical wound.

## Introduction

Glaucoma in aphakia may present months to years after paediatric cataract surgery, younger age at surgery being one of the risk factors for development of glaucoma [1]. It is one of the most difficult glaucoma to control with either antiglaucoma medications or with conventional glaucoma filtering surgeries [2]. Implantation of glaucoma drainage device provides an effective approach to manage refractory paediatric glaucoma [2]. However implant surgery in young individuals is not free of complications. The common adverse events reported in literature are hypotony, choroidal detachment, tube-cornea touch and tube erosion [3]. The prompt detection and management of tube erosion is of utmost importance to prevent devastating sequel of endophthalmitis [4]. Re-erosions have been reported and hence need meticulous long term follow-up. Implantation cyst following repair of tube erosion has not been reported so far. In this report, we have discussed the possible causes, the sequel, its prevention and management.

## Case description

**Figure 1:**
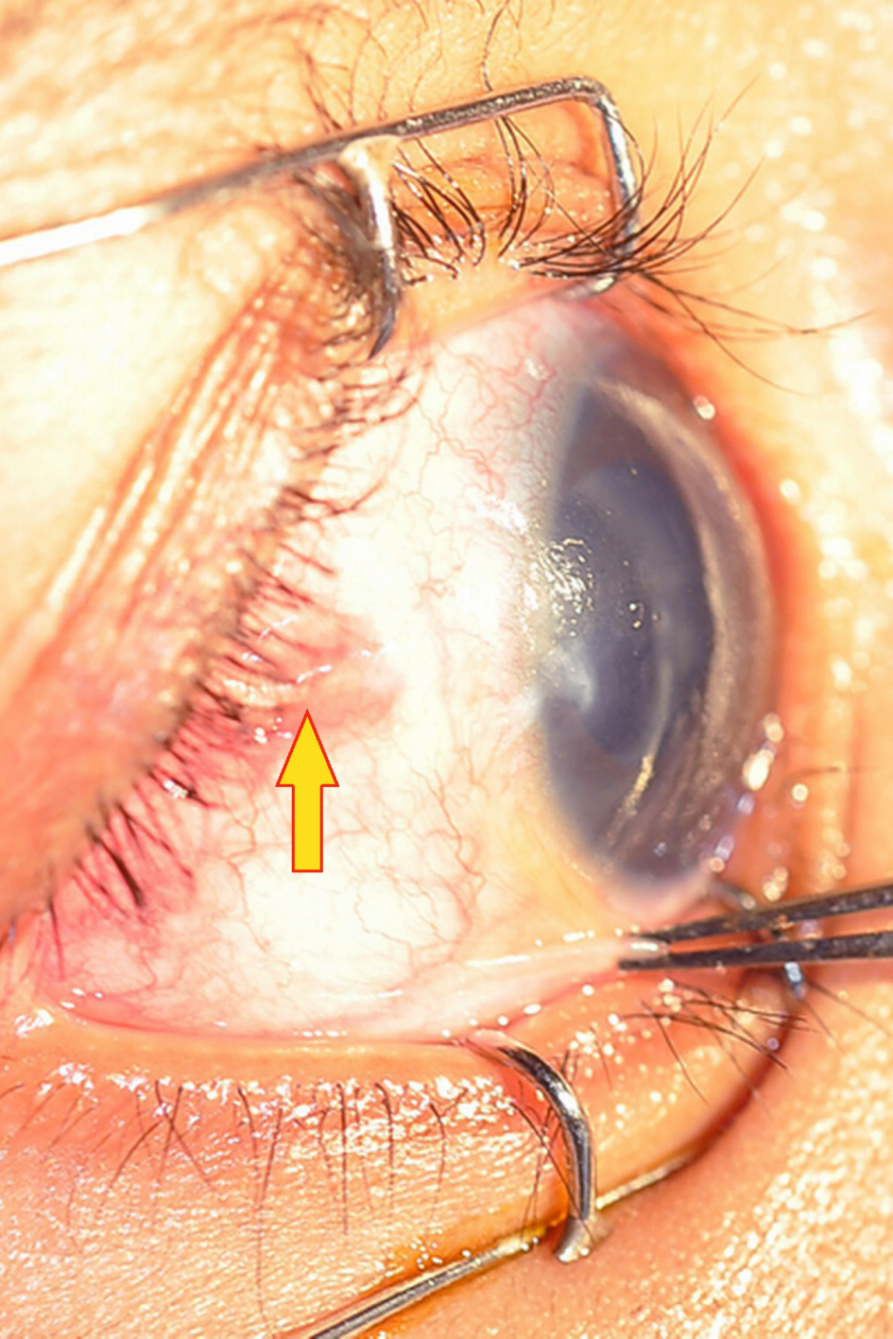
Tube erosion

**Figure 2:**
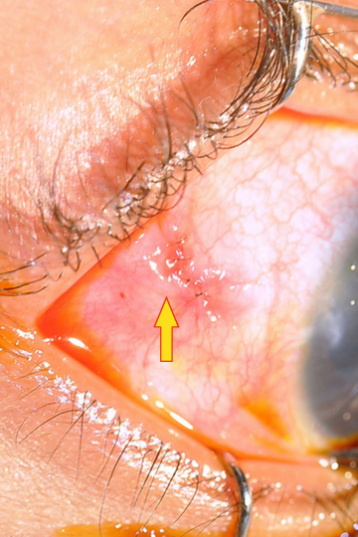
Conjunctival closure

**Figure 3:**
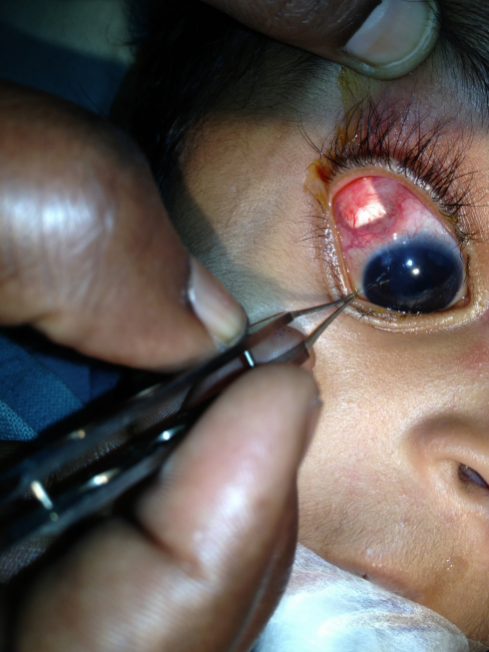
Conjunctival inclusion cyst

**Figure 4:**
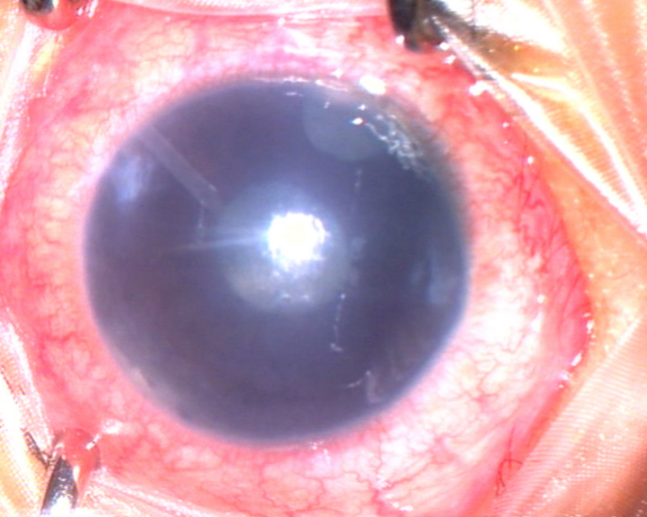
Endophthalmitis

**Figure 5:**
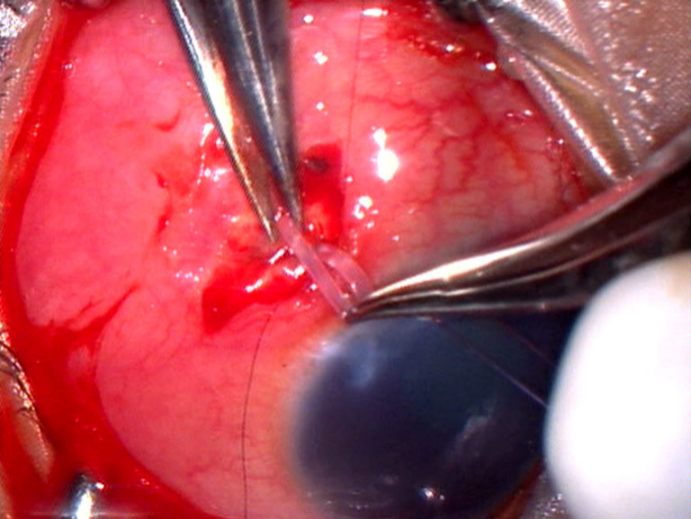
Tube excision

**Figure 6:**
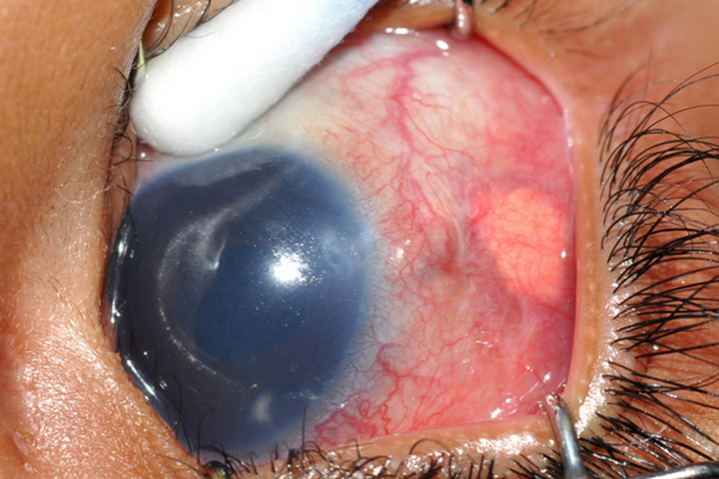
Final profile

A 2-year-old boy with glaucoma in aphakia and failed previous combined trabeculotomy and trabeculectomy had elevated intraocular pressures of 22 mgHg in right eye and 26 mmHg in left eye, on three antiglaucoma medications in both eyes. He underwent sequential paediatric AGV implantation in both the eyes at five months apart. The intraocular pressures at first postoperative month were 8 mmHg in right eye and 10 mmHg in left eye. Six weeks after the implant surgery, the right eye developed an erosion of conjunctiva overlying the tube with resorption of the scleral patch graft (Figure 1 (Fig. 1)). The erosion was initially managed with simple closure of conjunctiva with 10-0 nylon (Figure 2 (Fig. 2)). But he developed re-erosion ten days later. He was then treated with scleral patch graft and conjunctival advancement. Two and half months later, the child presented with an elevated lesion (inclusion cyst – Figure 3 (Fig. 3)) in the superotemporal quadrant over the area of scleral patch graft. The conjunctival inclusion cyst was aspirated with a 29G needle at the edge of the cyst to avoid any trauma to the overlying conjunctiva. However, he presented 3 months later following an episode of bilateral conjunctivitis with endophthalmitis (Figure 4 (Fig. 4)). There were yellow glow from fundus with exudates in the vitreous and the tube. He underwent an emergency pars plana vitrectomy with intraocular antibiotics and tube excision (Figure 5 (Fig. 5)). The endophthalmitis resolved with appropriate treatment however, the IOP increased to 36 mmHg on three antiglaucoma medications. The IOP control was achieved with transscleral cytophotocoagulation procedure and two antiglaucoma medications and was maintained at 20 mmHg until the 18^th^ month follow up at the time of writing this manuscript (Figure 6 (Fig. 6)).

## Discussion

The inclusion cysts are the commonest cystic lesions of conjunctiva. They develop from the embedding of conjunctival epithelial cells into the deeper layer as a result of surgery or trivial trauma such as eye rubbing. Acquired conjunctival inclusion or implantation cysts have been reported following small incision cataract surgery, retinal detachment surgery, squint surgery, corneal procedures etc [5], [6], [7]. During the primary implantation of Ahmed glaucoma valve we routinely cover the tube with the help of donor cadaveric scleral patch graft and fibrin glue. In this case it is possible that the scleral patch graft was displaced due to constant eye rubbing [8] and the underlying sutures were exposed eroding the conjunctiva. Fibrin glue is a strong sealant; however it undergoes fibrinolyis by about two weeks. We expect secure graft adhesion by this time. It would be a better option to place extra sutures to secure the patch graft in addition to the glue in children to prevent graft displacement and consequent tube erosion. This highlights the fact that one should take extra precaution while counseling the parents against eye rubbing of their children e.g. to use eye shield at bed time etc. Also it is important to keep the threshold of extensive glaucoma filtering surgery like valve implant in children relatively high because of the potential to cause more sinister complications. In our case we hypothesize that the inclusion cyst could have developed due to trauma during repeated surgeries and associated inflammation or it was from the epithelial lining underneath the eroded tube which was not debrided during the surgery. The inclusion cyst, when formed, has various treatment options described in the literature such as complete excision of the cyst, puncture of the cyst and aspiration of the contents, partial excision of the cyst, trichloracetic acid injection into the cyst etc. In consultation with our oculoplasty team we preferred to aspirate the cyst rather than completely excise it as sacrificing the conjunctiva would be difficult. There was no recurrence of the cyst however, the child developed endophthalmitis due to recurrent erosion at a later date. The erosion was not in the same area as the site of cyst aspiration, this helps us to postulate that the stretched conjunctiva with constant eye rubbing would have led to thinning of the tissue at a different place and recurrent erosion. These sequences of events indicate that simple conjunctival closure over an area of tube erosion is not going to survive for long. Hence during the repair of tube erosion, it is very important to carefully deepithelize the area under and around the tube by gentle cautery/ scraping before placing the patch graft to avoid epithelial inclusion cyst. It would also be beneficial to minimize tension in the tissue during repair by using conjunctival autograft, thereby reducing the risk or re-erosion and the fatal sequel of endophthalmitis.

## Notes

### Competing interests

The authors declare that they have no competing interests.
